# A unilateral robotic knee exoskeleton to assess the role of natural gait assistance in hemiparetic patients

**DOI:** 10.1186/s12984-022-01088-2

**Published:** 2022-10-08

**Authors:** Julio Salvador Lora-Millan, Francisco José Sanchez-Cuesta, Juan Pablo Romero, Juan C. Moreno, Eduardo Rocon

**Affiliations:** 1 Centro de Automática y Robótica, Consejo Superior de Investigaciones Científicas - Universidad Politécnica de Madrid, Madrid, Spain; 2 Electronic Technology Department, Universidad Rey Juan Carlos, Madrid, Spain; 3 Facultad de Ciencias Experimentales, Universidad Francisco de Vitoria, Pozuelo de Alarcón, Madrid, Spain; 4 Brain Damage Unit, Hospital Beata María Ana, Madrid, Spain; 5 Neural Rehabilitation Group, Cajal Institute, Spanish National Research Council (CSIC), Madrid, Spain

**Keywords:** Hemiparetic gait, Stroke patients, Robotic exoskeleton, Unilateral assistance, Gait symmetry

## Abstract

**Background:**

Hemiparetic gait is characterized by strong asymmetries that can severely affect the quality of life of stroke survivors. This type of asymmetry is due to motor deficits in the paretic leg and the resulting compensations in the nonparetic limb. In this study, we aimed to evaluate the effect of actively promoting gait symmetry in hemiparetic patients by assessing the behavior of both paretic and nonparetic lower limbs. This paper introduces the design and validation of the REFLEX prototype, a unilateral active knee–ankle–foot orthosis designed and developed to naturally assist the paretic limbs of hemiparetic patients during gait.

**Methods:**

REFLEX uses an adaptive frequency oscillator to estimate the continuous gait phase of the nonparetic limb. Based on this estimation, the device synchronically assists the paretic leg following two different control strategies: (1) replicating the movement of the nonparetic leg or (2) inducing a healthy gait pattern for the paretic leg. Technical validation of the system was implemented on three healthy subjects, while the effect of the generated assistance was assessed in three stroke patients. The effects of this assistance were evaluated in terms of interlimb symmetry with respect to spatiotemporal gait parameters such as step length or time, as well as the similarity between the joint’s motion in both legs.

**Results:**

Preliminary results proved the feasibility of the REFLEX prototype to assist gait by reinforcing symmetry. They also pointed out that the assistance of the paretic leg resulted in a decrease in the compensatory strategies developed by the nonparetic limb to achieve a functional gait. Notably, better results were attained when the assistance was provided according to a standard healthy pattern, which initially might suppose a lower symmetry but enabled a healthier evolution of the motion of the nonparetic limb.

**Conclusions:**

This work presents the preliminary validation of the REFLEX prototype, a unilateral knee exoskeleton for gait assistance in hemiparetic patients. The experimental results indicate that assisting the paretic leg of a hemiparetic patient based on the movement of their nonparetic leg is a valuable strategy for reducing the compensatory mechanisms developed by the nonparetic limb.

## Background

Stroke is the second most common cause of death in Europe [1] and one of the leading causes of long-term disability worldwide [1, 2]. Due to population aging and improved survival rates, it is estimated that the number of people living with stroke will increase by 27% between 2017 and 2047 in the EU [3]. Of those who survive a stroke, 80% present motor dysfunction [4, 5], and 65% present gait impairment [6] that affects their independence and quality of life [7, 8] and hampers the performance of daily life activities [9].

Hemiparetic gait is the most common gait disturbance due to stroke, and it is characterized by a strong asymmetric gait pattern resulting from contralateral motor weakness, motor control deficits, sensory and/or proprioceptive loss, and/or ataxia [10]. This asymmetrical gait supposes poor single limb support and uncontrolled forward movement [11]. Consequently, the nonparetic limb develops adaptations to compensate for this misfunction [12].

For example, the swing phase in the nonparetic leg is shortened, and the stance phase is lengthened compared with the paretic limb, which leads to temporal asymmetry [9]. Step length asymmetry is highly variable in stroke patients and representative of different compensatory strategies, and in patients with symmetric step length, it does not necessarily imply symmetric forward propulsion [13]. Other typical compensation mechanisms are pelvic hike or circumduction to overcome reduced foot clearance during the paretic swing [12].

Despite physical therapy, which can improve the speed and endurance of hemiparetic subjects, asymmetric gait can be resistant to intervention [14] and is still present in 50% of community-dwelling chronic stroke patients [15]. Hemiparetic gait might cause several consequences such as musculoskeletal pathologies in the nonparetic limb, falls due to instability, slow gait velocity, or increased energy consumption [15, 16]. It is also an important factor for people with stroke, who are concerned about walking appearance [13, 17].

In this context where motor recovery after stroke remains a clinical challenge [18], robotic exoskeletons have been presented not only as rehabilitation tools [19] but also as assistive devices for hemiparetic subjects. Several authors have developed robotic devices to improve poststroke gait quality, and some of them opted for bilateral configurations to assist and/or measure both paretic and nonparetic legs, such as the HAL exoskeleton [20, 21], or the Curara prototype [22].

Conversely, other authors adopted unilateral configurations for their approaches, meaning that the device was worn only on the paretic leg. These devices present several advantages as they are simpler and lighter than bilateral devices [23], and they can target the specific function of the affected joint during gait [24]. For example, the commercial C-Brace Orthotronic Mobility System from Otto Bock HealthCare, is an active KAFO that assists knee flexion and extension during swing and stance and has been demonstrated to improve the gait pattern of hemiparetic patients [25, 26], although their hemiparesis was not a consequence of stroke but of other conditions. These patients were able to reduce joint compensatory peak movements to normal levels [25], validating that this device facilitates the execution of activities of daily living compared to conventional KAFOs [26].

However, the unilateral configuration of a robotic exoskeleton implies that the controller needs to manage the appropriate coordination between the movements of the nonassisted joints and the assisted ones. For example, the actions of the Tibion robotic knee orthosis [27] or the Soft Ankle Exosuit from Harvard University [28, 29] are based on detecting gait events and reported improvements in the gait symmetry of stroke patients. The ALEX II exoskeleton also improved the gait symmetry in poststroke subjects [30], although its action followed a force field around a healthy foot trajectory, thus exploiting the interjoint coupling to coordinate the assistance.

Previous research also explored the use of the movement of the nonparetic leg to coordinate the action of assistive robotic exoskeletons. For example, some authors considered that the movement of one leg can be used to estimate the movement of the contralateral leg and applied an assistive strategy or directly replicated the gait pattern of the nonassisted leg [20, 21, 31–34]. The prototype developed by Peng et al. also based its movement on sound limb kinematics, but according to a leader–follower multiagent system framework [35]. Instead of directly using the trajectory depicted by the unimpaired leg, the desired position of the impaired limb can be estimated according to the position of the unimpaired limb. Following this approach, the complementary limb motion estimation strategy computed the paretic limb’s desired joint positions based on the current position of the nonparetic limb by using synergetic information from healthy reference subjects [36–38].

Although the studies mentioned above have demonstrated the feasibility of robotic assistance to hemiparetic gait, several aspects have not yet been fully addressed, such as the users’ adaptation to this assistance or the differences between assistive strategies. In this article, we present the robotic exoskeleton REFLEX (symmetRy rEinForcer uniLateral powEred eXoskeleton), a prototype developed as a tool to investigate these adaptation processes and the effect of unilateral gait assistance on hemiparetic subjects. This device was designed to assist the motion of the paretic knee of stroke survivors to promote a symmetric gait by guiding knee movement during the swing phase and reinforcing the knee joint during the stance phase of the gait, as was previously reported to be a successful target [27, 39]. In this framework, we defined two different strategies to control the robotic exoskeleton based on the motion of the unassisted leg: (1) replicating the movement of the nonparetic limb or (2) synchronically applying a healthy gait pattern to guide the movement of the limb.

The general objective of developing these strategies is to enable the intuitive control of the device that improves the embodiment of the technology by enabling a natural interaction between the user and the robotic exoskeleton. This embodiment would imply that the users assimilate the device’s movement as generated by their own body instead of being generated by an external tool [40]. We hypothesize that the proper and natural interaction between humans and devices will enable patients to consider the robot’s action as a part of their own gait capability, improving their gait quality as a consequence. Hemiparetic asymmetry is not only due to impairments in the affected limb, but it is also the consequence of biomechanical compensatory mechanisms that might arise in the nonparetic leg. The REFLEX prototype thus serves as a tool to assess the adaptation process of the subject to the exoskeleton assistance and to evaluate the effects of such human–robot interaction in both paretic and nonparetic legs.

## Methods

### Robotic exoskeleton design

**Fig. 1:**
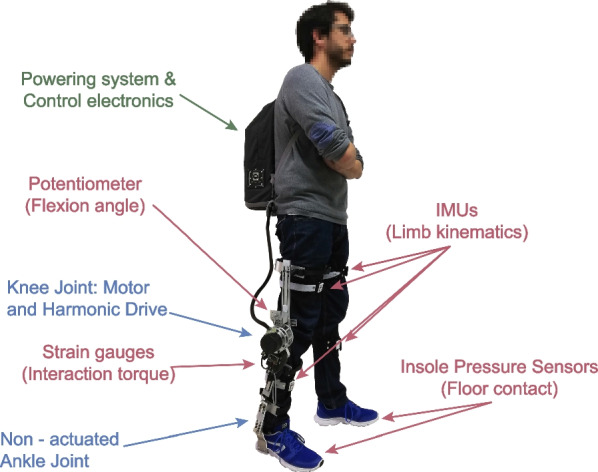
REFLEX prototype for the assistance of the knee joint of the paretic leg. This joint is actuated by a DC motor coupled to a Harmonic Drive while the ankle remains unactuated. The sensors of the prototype are a potentiometer to measure the exoskeleton flexion in the sagittal plane, strain gauges to measure the interaction torque, inertial sensors (IMUs) to acquire the lower-limbs kinematics, and insole pressure sensors to detect floor contact events

The REFLEX prototype is a knee–ankle–foot orthosis (KAFO) composed of two joints aligned to the knee and ankle of the user (Fig. 1). The segments lengths and the braces positions can be tailored to the anthropometry of different users. The material of the major part of the prototype is aluminum 7075, so the result is a robust and lightweight device.

The knee joint is actuated by a DC EC-60 flat 408,057 brushless motor (Maxon ag, Switzerland) coupled with a CSD-20-160-2AGR harmonic drive (Harmonic Drive LLC, EE.UU.). The transmission ratio of 1:60 of this system enables the application of a mean torque of 35 Nm, which is required to perform the limb movement [41]. The ankle joint of the prototype remains nonactuated and unconstrained, enabling its free movement in the sagittal plane. The total weight of the KAFO is approximately 4 kg. The prototype is equipped with four sets of sensors that provide information on system variables that are used for the control in real-time:

1. A potentiometer, coupled with the joint axis, is used to measure the flexion/extension angle of the active joint. This information enables the robot to follow trajectories using a closed-loop position control algorithm.
2. An interaction torque sensor is placed between the robot and the user. It consists of two pairs of strain gauges in a full Wheatstone bridge. This interaction torque is used to implement an impedance controller that adjusts the torque provided by the robotic system to the user’s leg. This controller is fully described in the subsection "Variable impedance low-level controller".
3. Three insole pressure sensors based on FSRs (Force Sensing Resistors) are used to assess the contact of each user’s foot with the floor. These measurements distinguish between swing and stance phases and adapt the controllers accordingly.
4. Four Inertial Measurement Units (IMUs) (TechMCS, Technaid, Spain) are used to compute the kinematics of both legs. They are attached to the shanks and thighs of both legs ensuring a proper alignment between the anatomical and sensors axes, and are used to measure the flexion/extension angles of the hips and knees [42].

 

The control system is based on the LaunchXL-F38377S board (Texas Instrument, USA), which runs the control algorithm and acquires sensor data at 1 kHz (except for IMUs, whose sample frequency is 50 Hz). The system can be used in a tethered version or a as portable setup where the exoskeleton’s control electronics and a Li-Po battery are embedded in a backpack that the user carries. The total weight of the power and electronic system is approximately 3 kg.

### Gait assistive control strategies for the REFLEX prototype

**Fig. 2:**
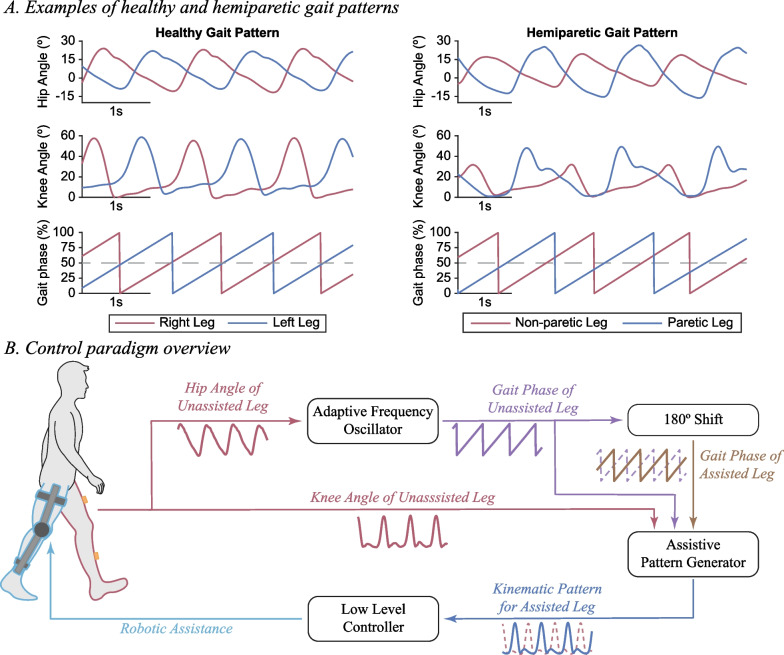
Control paradigm. **A** Examples of healthy and hemiparetic gait patterns. **B** An overview of the control algorithm. The assistance provided by the robotic exoskeleton is synchronized with the movement of the unassisted leg. The unassisted hip angle feeds an adaptive frequency oscillator to estimate the unassisted leg’s gait phase in realtime. This phase is shifted 180° to obtain the gait phase for the assisted leg. The gait phase of both legs and the unassisted knee movement are used to generate the pattern to be followed by the exoskeleton through the low-level controller to assist the movement of the assisted limb

The main aim of the REFLEX prototype is to deliver assistance to the paretic leg of hemiparetic subjects according to the movement of the nonparetic leg. As an example, Panel A of Fig. 2 shows the gait pattern of both lower limbs for a healthy subject and a hemiparetic patient. Although there are few differences in the functional gait pattern of both limbs in the healthy subject (as reported by Sadeghi et al. [43]), these differences are more significant in the hemiparetic patient. In Panel A of Fig. 2, we also represent the continuous gait phase, which is a function that increases monotonically from 0 to 100% between consecutive heel strikes. As reported in previous works, the phase of the movement of both limbs is shifted approximately 180º in healthy subjects [44, 45].

Our control approach uses the information of the nonparetic leg measured by the corresponding IMUs to assist the paretic leg synchronically (Fig. 2, Panel B). This approach is implemented in three phases. First, the synchronization is based on the real-time gait phase estimated by an adaptive frequency oscillator (AO) [46], a mathematical tool that is synchronized with the nonparetic hip angle by learning its features as variable states. Second, we estimate the desired gait phase of the assisted leg by shifting 180° the real-time gait phase calculated by the AO and the assistive gait pattern based on the kinematics of the nonparetic leg. Third, the low-level controller generates a force-tunnel around this desired kinematics pattern to assist the limb.

#### Real-time gait phase estimation

We use an AO to estimate the real-time gait phase and thus synchronize the REFLEX prototype with the user’s healthy side movement [47, 48]. Compared with other published control paradigms that synchronize the robot’s action, such as Finite-State-Machines (FSM) [49–52] or EMG-based controllers [53–56], AOs present several advantages. Compared with FSM controllers, AOs generate continuous signals instead of discrete trigger events, enabling more versatile strategies and the use of these signals in low-level controllers. In addition, compared with EMG controllers, the required sensory system is simpler and more robust.

AOs are dynamic systems that can be synchronized to periodic signals by learning their features as state variables [46]. Considering the hip flexion angle of the unassisted leg, which is measured by the thigh IMU, $\theta_{u}$, as input, an AO can estimate the phase of the unassisted leg $\varphi_{u} \left( t \right)$ according to the next dynamic system [47]:

1 $$\varepsilon \left( t \right) = \theta_{u} \left( t \right) - \hat{\theta }_{u} \left( t \right)$$

2 $$\dot{\omega } = - \nu_{\omega } \varepsilon \left( t \right)\sin \varphi_{u}$$

3 $$\dot{\varphi }_{u} = \omega - \nu_{\varphi } \varepsilon \left( t \right)\sin \varphi_{u}$$

4 $$\dot{\alpha }_{k} = \eta \cos \left( {k\varphi_{u} } \right)\varepsilon \left( t \right)\quad \left( {k = 0, \ldots ,N_{f} } \right)$$

5 $$\dot{\beta }_{k} = \eta \sin \left( {k\varphi_{u} } \right)\varepsilon \left( t \right)\quad \left( {k = 0, \ldots ,N_{f} } \right)$$

6 $$\hat{\theta }_{u} = \sum\limits_{k = 0}^{{N_{f} }} {\alpha_{k} \cos \left( {k\varphi_{u} } \right) + } \beta_{k} \sin \left( {k\varphi_{u} } \right)$$

where $\varphi_{u}$ and $\omega$ are the phase and frequency of the oscillator synchronized with the unassisted leg; $\alpha_{k}$ and $\beta_{k}$ are the Fourier coefficients used for estimating $\hat{\theta }_{u}$; and $\varepsilon \left( t \right)$ is the error in this estimation. $\nu_{\omega }$ and $\nu_{\varphi }$ are learning constants and $\eta$ is a coupling factor that determine the dynamic response of the error $\varepsilon \left( t \right)$. At every time step, a new input $\theta_{u}$ is considered, and it is used to update all the variables involved in the AO.

The gait phase $\varphi_{u}$ should be a variable that increases monotonically and is reset when the gait cycle is completed. However, the convergence of the AO may not fulfill the criteria of $\varphi_{u} = 0$ at heel strike. A phase offset correction is introduced to ensure that $\hat{\varphi }_{u} = 0$ at heel strike and give kinematic meaning to the gait phase estimation. According to [57], a phase correction was implemented, so an offset in the phase estimation $\rho$ is updated every time the insole pressure sensors detect the heel-strike event. This offset is low-pass filtered with a first-order Butterworth filter with a cutoff frequency of 0.5 Hz to avoid abrupt changes in the phase estimation due to phase correction.

7 $$\rho = \varphi_{{s_{HeelStrike} }}$$

8 $$\hat{\varphi }_{u} = \varphi_{u} - \rho$$

Once the AO estimates the corrected phase of the unassisted leg, we calculate the phase of the assisted leg $\varphi_{a}$ by shifting it $\pi$ rad according to the following equation.

9 $$\varphi_{a} = \hat{\varphi }_{u} + \pi$$

For convenience to gait analysis, the following changes of variables are performed:

10 $$\phi_{u} \left( t \right) = \frac{{\hat{\varphi }_{u} \left( t \right)}}{2\pi } \cdot 100$$

11 $$\phi_{a} \left( t \right) = \frac{{\varphi_{a} \left( t \right)}}{2\pi } \cdot 100$$

12 $$f\left( t \right) = \frac{\omega \left( t \right)}{{2\pi }}$$

By doing so, the gait phases $\phi_{u} \left( t \right)$ and $\phi_{a} \left( t \right)$ are maintained within the range of 0–100%, and they indicate the real-time percentage within the step. Likewise, the gait frequency $f\left( t \right)$ is the real-time frequency in steps per second.

#### Assistive pattern generators

Based on the estimation of the gait phase of both legs, the prototype generates an assistive pattern to render a given trajectory to the paretic leg that should contribute to improving gait symmetry. This reference trajectory also depends on the motion of the unassisted leg’s motion acquired in real-time by the corresponding IMUs.

We developed two different assistive strategies, namely, the Echo-control strategy, which aims to replicate the average movement of the unassisted leg, and the Adaptive healthy pattern strategy, which aims to synchronize the application of a healthy reference from the literature.

##### A. Echo-control assistive strategy

**Fig. 3:**
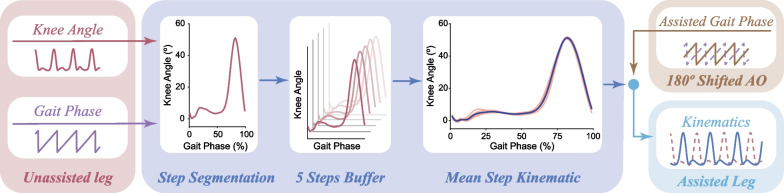
Pattern generation according to the Echo-control assistive strategy based on replicating the kinematics of the unassisted leg. The knee movement during a step is stored in a five-step buffer and used to calculate the mean pattern of the unassisted knee; afterward, this averaged movement is provided as the targeted reference for the robotic exoskeleton according to the gait phase estimated for the assisted limb

Figure 3 illustrates the concept of the Echo strategy, which aims at replicating the movement of the unassisted leg of the user [58]. In the first stage, we consider the movement of the unassisted leg, so the knee flexion/extension angle is mapped over the gait phase estimated by the AO. By using linear interpolators, the system reconstructs the step kinematics regularly separated by 2% of the step cycle. This information is stored in a buffer that retains the kinematics of the last five steps; thus, the average unassisted pattern is calculated as the mean of this buffer’s content.

The average kinematic pattern is smoothed by using a fifth-order zero-lag Butterworth filter designed for a sampling frequency of 50 Hz (the sampling frequency of the signal if the step has a duration of 1 s) and a cutoff frequency of 10 Hz (the fifth part of the sampling frequency). Once the signal is smoothed, it is derived to yield the velocity and acceleration patterns for the flexion movement, according to the following equations:

13 $$\dot{\theta }_{ref} = \frac{{d\theta_{ref} }}{dt} = \frac{{d\theta_{ref} }}{{d\phi_{a} }}\cdot\frac{{d\phi_{a} }}{dt}$$

14 $$\ddot{\theta }_{ref} = \frac{{d^{2} \theta_{ref} }}{{dt^{2} }} = \frac{{d^{2} \theta_{ref} }}{{d\phi_{a}^{2} }} \cdot \left( {\frac{{d\phi_{a} }}{dt}} \right)^{2}$$

The instantaneous values for $\theta_{ref}$, $\dot{\theta }_{ref}$ and $\ddot{\theta }_{ref}$ are calculated by linearly interpolating the current desired gait phase for the assisted leg between the points that define the three kinematic patterns (angle, velocity, and acceleration).

##### B. Adaptive healthy pattern assistive strategy

**Fig. 4:**
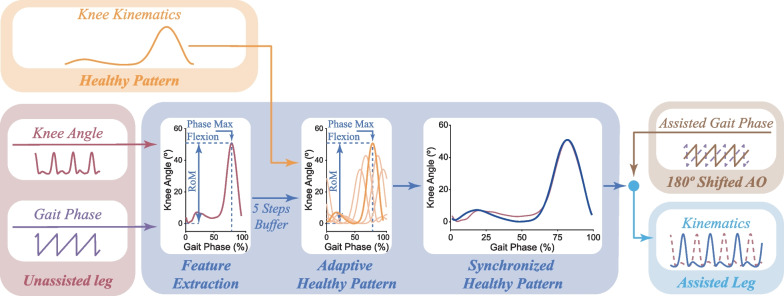
Assistive pattern generation based on synchronizing a healthy gait pattern. The knee pattern is scaled and shifted according to the features extracted from the movement of the unassisted leg; afterward, it is provided as the set point for the robotic exoskeleton according to the gait phase estimated for the assisted limb

The healthy pattern strategy uses the information of the unassisted leg to adapt a reference trajectory to the user’s movement; see Fig. 4. This pattern is generated based on the joint reference trajectory for robotic gait support published by Koopman et al. [59].

This strategy analyzes the kinematic pattern of the unassisted leg to extract the range of motion and the phase of maximum knee flexion during each step. These features are stored in a five-step buffer, so the average features of the last five steps are used to scale and shift the reference pattern. First-order low-pass Butterworth filters with a cutoff frequency of 0.5 Hz smooth these factors prior to their application to avoid abrupt changes.

Similar to the previous strategy, velocity and acceleration references are also generated. In this case, the first and second derivatives of the angular healthy pattern with respect to the gait phase are computed offline, and, afterward, they are scaled and shifted by the same factors as the angle reference. Applying Eqs. (13) and (14), these derivatives with respect to the gait phase are changed to the time domain to be used as velocity and acceleration references for the assisted leg. Once these reference patterns are fully defined, cubic splines are used to interpolate the values for $\theta_{ref}$, $\dot{\theta }_{ref}$ and $\ddot{\theta }_{ref}$ according to the desired gait phase for the assisted movement.

#### Variable impedance low-level controller

The assistance provided by the exoskeleton is based on a variable impedance model that aims to control the interaction between the robot and wearer [60]. As in previous related works [39, 61, 62], the controller used in the prototype has a twofold objective depending on the current gait phase:

1. During the stance phase, the robot aims to reinforce the limb so that the system composed of the leg and the exoskeleton can load the user’s weight and not collapse. A high impedance model is responsible for this reinforcement since it avoids substantial deviations from the assistive kinematic pattern.
2. During the swing phase, the robot guides the limb’s movement according to the assisted-as-needed (AAN) paradigm. The computed error between the reference kinematics and the actual movement serves as the input for the impedance model that defines the torque applied by the device to assist the user gait. No torque is applied if the error is null, while higher errors correspond to greater assistive torques. The impedance model depends on the assistance level selected by the therapist or the user, so the force tunnel around the desired trajectory can be changed according to the user’s needs. This assistance can vary from 0 to 100%, where an assistance of 0% commands the robot to not interfere with the user’s movement while an assistance of 100% does not allow the user to deviate from the prescribed trajectory.

**Fig. 5:**
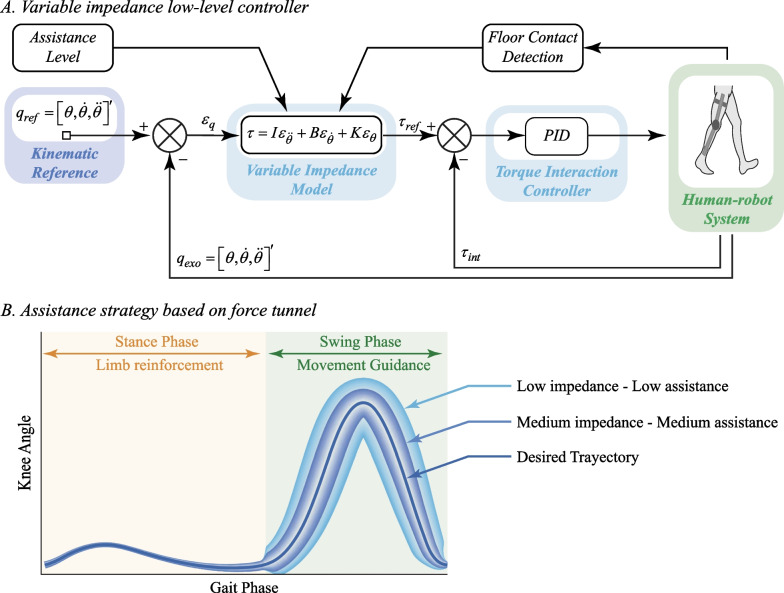
Low-level controller of REFLEX. **A** The block diagram of the variable impedance controller; this controller assists the knee movement following the kinematic reference and according to an Assisted-As-Needed paradigm. **B** The two assistance strategies followed during a single step: the exoskeleton reinforces the joint during the stance phase while it guides the movement during the swing phase; following the Assisted-As-Needed paradigm, the exoskeleton is able to provide different assistance levels by using different force tunnels as depicted in the image

According to the force-tunnel paradigm, the impedance model calculates the interaction torque that the exoskeleton should provide due to the angular reference tracking error. The system uses a PID controller to follow this torque interaction reference and provide it to the user (Fig. 5).

### Experimental validation

The validation of the REFLEX prototype was implemented in 3 phases. First, we implemented the technical validation of the controller’s performance, assessing the phase estimation provided by the AO and the pattern generated by the two assistive strategies. Then, in the second phase, we tested the device with healthy subjects to ensure its proper operation during human interaction. Finally, in the third phase, the prototype was evaluated with stroke patients.

In total, six volunteer subjects participated in the experiments. We recruited three healthy subjects (3 males, age: 24. 7 ± 3.8 years, height: 1.78 ± 0.02 m, weight: 77.7 ± 2.5 kg; mean ± standard deviation) and three chronic stroke patients (demographic data are summarized in Table 1). The patients had no cognitive impairment according to the Mini-Mental State Examination (MMSE). All of them required a trekking stick to shift medium and long distances and presented a modified independency according to the Functional Independence Measurement Scale (FIM).

**Table 1:** Stroke subjects’ demographic data

Ident	P1	P2	P3	Average^1^
Age (years)	63	57	54	58 ± 4.6
Height (m)	1.8	1.72	1.72	1.74 ± 0.05
Weight (kg)	83	84	60	75.6 ± 13.6
Time after stroke (months)	11	7	9	9 ± 2
Sex	Male	Male	Male	
Hemiparetic side	Left	Right	Right	
Stroke	Cortical ischemic	Subcortical hemorrhagic	Cortical ischemic	
Degree of dependency	Without help	Without help	Without help	
Functionality level	Modified independency	Modified independency	Modified independency	
Walking aid	Trekking stick	Trekking stick	Trekking stick	

^1^Mean ± standard deviation

All subjects gave their informed consent for the experiment; the study was conducted in accordance with the Declaration of Helsinki, and it was approved by the local ethics committee. All subjects were also instructed to walk on a treadmill at a constant speed during trials of 5 min each, and they carried out four different kinds of trials: (1) NoExo: subjects only wore the inertial sensors and the insole pressure sensors to acquire their basal motion; (2) Free: subjects wore the exoskeleton although the actuator was mechanically decoupled, so it enabled the free movement of the knee; (3) Echo: the device provided gait assistance following the Echo-control strategy; and (4) Pattern: the device provided gait assistance following the Pattern strategy. During trials, subjects used the tethered version of REFLEX, so they only wore the robotic KAFO (carrying a total weight of 4 kg). Additionally, stroke patients wore a safety harness that did not support any weight. Prior to the execution of the trials, the gait velocity was self-selected to a comfortable level by the subjects.

Healthy subjects also performed a previous trial to assess the controller’s performance under variable gait speed (VariableSpeed). They walked over the treadmill as in the NoExo condition, although the gait speed randomly varied from 1 to 3 km/h in 0.2 km/h steps for at least 15 s.

Subjects rested between trials for at least 5 min to avoid adaptation and learning effects from trial to trial. In addition, in the NoExo, Free, Echo, and Pattern trials, only the last 2 min of the trials were processed to evaluate the gait once the steady state was reached. For the processing of the data, we considered the beginning of each step when the insole pressure sensors detected heel strikes. All experimental data were recorded at 50 Hz.

### Data analysis

To compare joint motion between the two conditions, we used the phase portraits of these movements. This representation shows the angular position in the X-axis and the angular velocity in the Y-axis, so the resulting portrait’s shape is representative of the dynamics of the motion [63]. We defined the next metric to evaluate the similarity between two phase portraits:

15 $$Similarity\left( \% \right) = \frac{A \cap B}{{A \cup B}} \cdot 100$$

where $A$ and $B$ are the areas of two phase portraits, so $A \cap B$ is the common area between them and $A \cup B$ is the union of both areas.

We also evaluated the symmetry of gait metrics by using the Symmetry Index (SI) introduced by Arazpour et al. [64]:

16 $$SI\left( \% \right) = \frac{{\overline{X}_{A} - \overline{X}_{U} }}{{\tfrac{1}{2}\left( {\overline{X}_{A} + \overline{X}_{U} } \right)}} \times 100$$

where $\overline{X}_{A}$ and $\overline{X}_{U}$ are the mean values for a metric in the assisted and unassisted leg, respectively. A SI of zero value indicates a complete symmetry, so a higher absolute value means a higher asymmetry in the metric. The sign of the SI is related to the leg that showed the highest value for the metric; if the assisted leg presents the highest average metric, the SI is positive, while SI is negative if the unassisted leg shows the highest value.

When data distributions were compared, they were composed of the metrics calculated in each step during an experimental condition. After checking the nonnormality of the data (Kolmogorov–Smirnov test; P < 0.005) and the heteroscedasticity (Levene test; P < 0.005), we looked for significant differences between experimental conditions (Kruskall–Wallis test; P < 0.005).

## Results

### Technical validation

For the technical validation of REFLEX, we evaluated its performance on healthy subjects. First, we assessed the estimation of the continuous gait phase and frequency performed by the AO; afterward, we generated the assistive patterns offline based on VariableSpeed and NoExo data and compared them with the actual movement of the subjects.

#### AO validation

We used the left hip angle and the left heel strike as inputs of our AO to estimate the real-time phase and frequency of the gait. We compared these results with the real gait phase and frequency computed offline according to heel strike events.

**Fig. 6:**
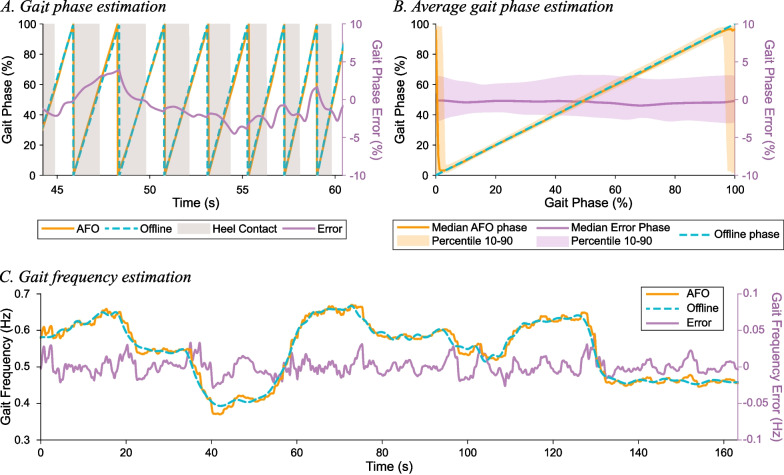
Example of AO results during the trial of one healthy subject walking at variable gait velocity. **A** Compares the AO phase estimation (solid orange line) with the offline phase calculated based on the heel strike detected by the insole pressure sensors (dashed cyan line) during a trial segment. **B** The median phase estimation during a step in the trial and median error during the step; areas represent the 10th–90th percentiles. **C** Compares the gait frequency estimated by the AO in real-time (solid orange line) with the gait frequency calculated offline based on heel strikes (dashed cyan line) during the trial. For all the panels, purple lines show the error between the AO estimation and the offline result and are represented with respect to the right axis

Figure 6 illustrates the AO estimations for one trial; Panels A and C show how the AO adapted its estimations to velocity changes. Table 2 summarizes the error for the phase and frequency estimated with the AO in all subjects. The experimental results indicated that the gait phase RMS error (RMSE) is lower than 2.4% and that the frequency RMSE is lower than 0.015 Hz.

**Table 2:** Errors in gait phase and frequency estimation

	Average error^1^	RMSE
Gait phase (%)	− 0.28 ± 2.36	2.37
Frequency (Hz)	− 2.16e−04 ± 0.014	0.014

^1^Mean ± standard deviation

#### Assistive pattern generation

**Fig. 7:**
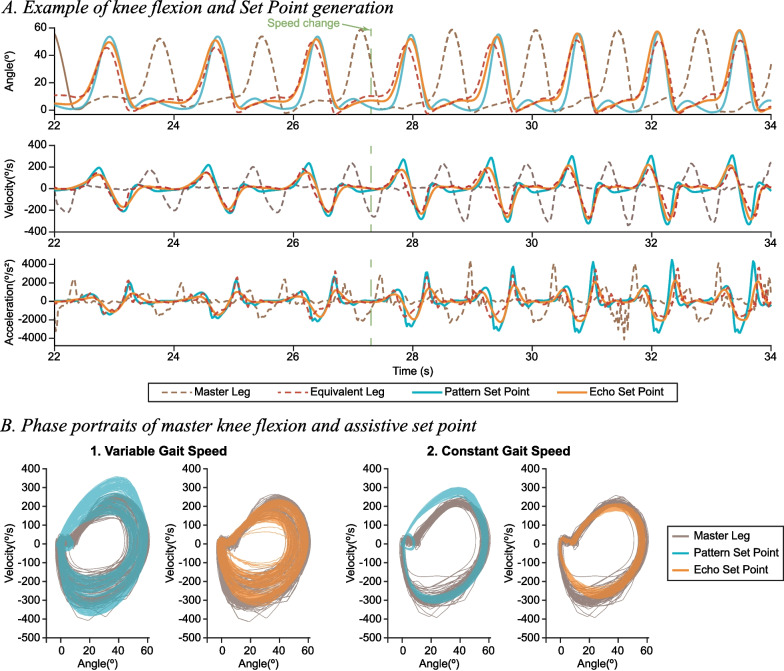
Example of set point generation with the experimental data of one healthy subject. **A** An example of the set-point calculated by the two assistive strategies (Pattern in blue and Echo in orange); they are compared with the movement of the master leg that fed the algorithm (brown dashed line) and with the movement of the equivalent leg whose motion should be synchronized with the generated set point (red dashed line). **B** Compares the phase portrait of the movement of the master leg (in brown) with the set point generated by the Pattern assistive strategy (in blue) or the echo assistive strategy (in orange). Two experimental conditions were evaluated: variable gait speed (two left panels) and constant gait speed (two right panels)

For healthy subjects, we considered the left leg as the master leg (corresponding to the nonparetic leg in hemiparetic subjects, i.e., the leg that is the reference for the pattern generation), so the generated reference should be synchronized with the movement of the right leg (also named the equivalent leg). Panel A of Fig. 7 shows the kinematic pattern generated by both assistive strategies during a portion of one VariableSpeed trial; it is remarkable how the controller reacted to gait velocity changes with minimal loss of synchrony.

**Fig. 8:**
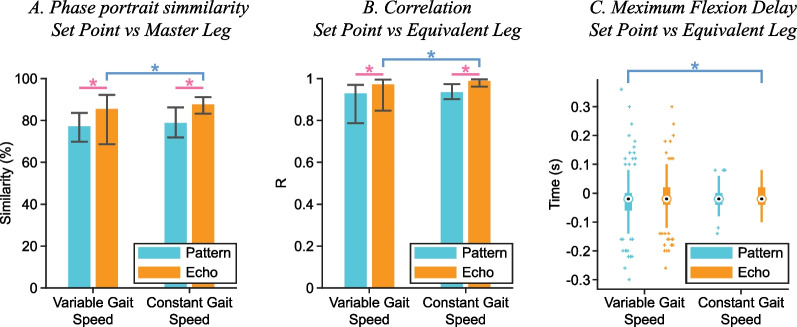
Assessment of the set-point generated by the two assistive strategies. **A** The phase portrait similarity between the set-point and the movement of the master leg used for generating it. **B** The correlation between the set-point and the movement of the equivalent leg (a correlation of 1 corresponds to a perfect synchronization). Length bars indicate the median value and whiskers indicate the 10th–90th percentiles. **C** The boxplot of the delays between the maximum knee flexion between the set point and the movement of the equivalent leg. Markers (*) show significant differences between experimental conditions

Panel B of Fig. 7 shows the phase portraits of the generated pattern and the master leg’s motion used as the basis. Note that during the trials at variable gait speed, the dispersion of the phase portraits is higher than for the trials at a constant speed. We adopted three metrics to evaluate the pattern generated with both assistive strategies, aiming to assess its waveform and timing:

1. Similarity between the generated pattern and the movement of the master leg. To evaluate this, we assess the similarity between the phase portraits. Panel A in Fig. 8 represents the distributions of this metric, and Table 3 summarizes the average value. The average similarity of the assistive pattern with the master leg’s motion is 77.5 ± 5.92% for the Pattern strategy and 84.31 ± 8.43% for the Echo strategy.
2. Timing of the generated pattern. To this end, we compared this pattern with the movement performed by the equivalent leg. We evaluated this timing in two ways: by checking the correlation between both movements and assessing the maximum knee flexion delay in both patterns. In Fig. 8, Panels B and C represent the distribution of these metrics, and Tables 4 and 5 summarize their average values. For the Pattern strategy, the average correlation is R = 0.9 ± 0.1, and the average delay is − 0.021 ± 0.060 s, while the Echo strategy resulted in a correlation of R = 0.95 ± 0.08 and an average delay of − 0.012 ± 0.053 s.

**Table 3:** Phase portrait similarity (%) between assistive pattern and master leg

	Pattern	Echo
Variable gait speed	77.03 ± 5.98	82.52 ± 10.03
Constant gait speed	78.57 ± 5.63	87.22 ± 3.40
Global	77.63 ± 5.89	84.35 ± 8.44

Mean ± standard deviation

**Table 4:** Correlation (R) between assistive pattern and equivalent leg

	Pattern	Echo
Variable gait speed	0.89 ± 0.12	0.94 ± 0.10
Constant gait speed	0.93 ± 0.03	0.98 ± 0.02
Global	0.90 ± 0.10	0.95 ± 0.08

Mean ± standard deviation

**Table 5:** Maximum flexion delay (s) between assistive pattern and equivalent leg

	Pattern	Echo
Variable gait speed	− 0.023 ± 0.071 (0.074)	− 0.015 ± 0.063 (0.065)
Constant gait speed	− 0.017 ± 0.037 (0.041)	− 0.007 ± 0.034 (0.035)
Global	− 0.021 ± 0.060 (0.064)	− 0.012 ± 0.053 (0.055)

Mean ± standard deviation (RMS error between brackets)

### Validation on healthy subjects

**Fig. 9:**
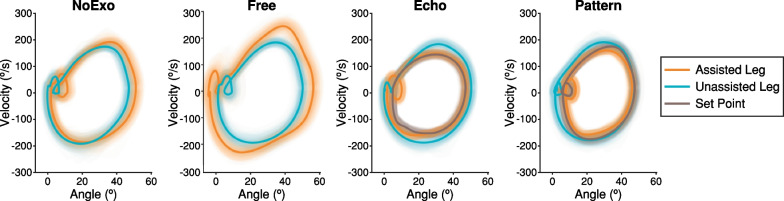
Phase portrait representation of the knee movement from the trials performed by a healthy subject (HS1). Orange lines correspond to the movement of the assisted knee while cyan lines correspond to the movement of the unassisted knee; brown lines correspond to the set point followed by the robot. Solid lines represent the median movement, while semi-transparent lines correspond to the movement of individual steps. When the user wears the robot, the phase portrait of the assisted leg changes in hip and knee joints; however, the action of the robot during Echo and Pattern conditions compensates for the effect of wearing the robot and makes the phase portrait of the assisted leg more closely approach to the portrait of the unassisted leg

**Fig. 10:**
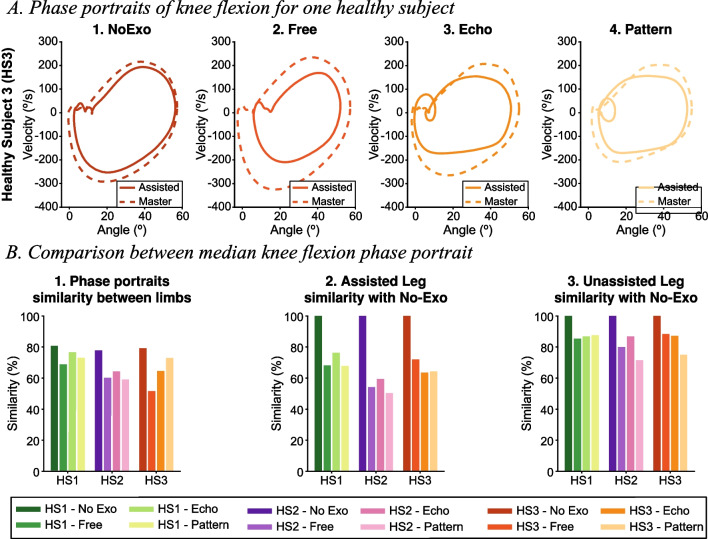
**A** The median phase portrait for each leg; Columns 1–4 include information for each experimental condition. Solid lines represent the assisted leg, and dashed lines represent the unassisted leg. **B** The similarity between the areas of the phase portrait: Panel **B1** represents the similarity between both limbs under each experimental condition, and Panels **B2** and **B3** represent the similarity between the movement under the current experimental condition and the movement during NoExo for each leg. Across the figure, data for the same healthy subject (HS) is represented in the same color (green for HS1, purple for HS2, orange for HS3) while the brightness indicates the experimental condition (from darkest to lightest: NoExo, Free, Echo, and Pattern). Similarity decreases because of wearing the robot; however, the assistance provided by the exoskeleton actively improves similarity in all patients

The following results are from the experiments that we conducted to understand how the assistance provided by the exoskeleton affects the gait of healthy subjects. Figure 9 illustrates, as an example, the knee phase portraits during one experimental trial with one healthy subject. Comparing how the phase portraits evolved across trials allows us to visualize how the movement of both knees (actuated and not) changed according to the REFLEX operation mode. Panel A in Fig. 10 shows the average phase portraits of both legs during trials and how the similarity measured in the NoExo trial decreased due to wearing the robot in the Free trial. However, the action of the robot actively improved similarity during the Echo and Pattern trials.

To evaluate the similarity between phase portraits, we used the metric defined in Eq. (15). Panel B of Fig. 10 shows the results of comparing different median phase portraits according to this metric. Panel B1 represents the comparison between assisted and unassisted motion in each experimental trial. The results indicate that the same behavior was replicated in all subjects. Wearing the device hampered the similarity between limbs, i.e., the symmetry; however, the provided assistance partially compensated for the effect of wearing the device since the similarity increased with respect to the Free condition. Nonetheless, it did not reach the similarity level of the NoExo condition. Only the Pattern strategy in Subject 2 did not improve the similarity with respect to the Free condition. Changes in interlimb similarity were due to variations in the assisted leg (Fig. 10, Panel B2), but also in the unassisted leg (Fig. 10, Panel B3).

**Fig. 11:**
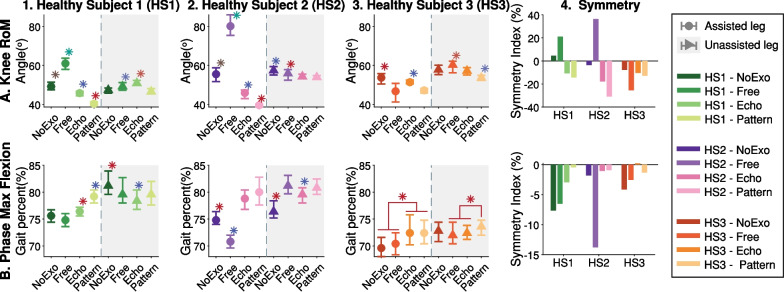
Knee kinematics symmetry for healthy subjects. Rows **A** and **B** represent the knee Range of Motion and the phase at maximum flexion for the knee. Columns 1–3 represent the result for each subject; markers indicate the median value and whiskers the 10–90 percentiles. Column 4 represents the symmetry index for each subject under each experimental condition; notice that symmetry indexes closer to 0 mean a higher symmetry between limbs. Across the figure, colors represent the same healthy subject (HS) (HS1 in green, HS2 in purple and HS3 in orange), the brightness represents the experimental condition (from darkest to lightest: No-Exo, Free, Echo and Pattern correspondingly) and the shape of the marker represents the assessed limb (circle for the assisted limb and triangle for the unassisted leg). Markers (*) show significant differences between experimental conditions within a limb of a subject

To evaluate the performance of the assistance, we also assessed the effect of the exoskeleton’s action over the range of motion (RoM) of the knee and the gait cycle percentage of the maximum knee flexion (Fig. 11). We considered the metric value in each step to create a distribution for each subject during each experimental condition.

The fourth column of Fig. 11 shows the analysis of the symmetry of these metrics. The results indicate that the RoM and the phase of maximum knee flexion were more symmetric during assisted trials than during the Free trials. However, although the highest symmetry for the phase of maximum knee flexion was achieved during assisted trials, the highest RoM symmetry was yielded during the NoExo trial.

### Validation on hemiparetic patients

**Fig. 12:**
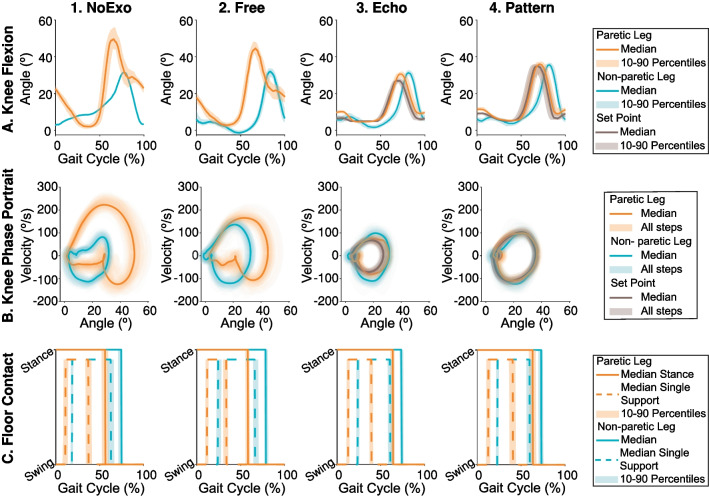
Example of step data from a hemiparetic patient (P2) during the different experimental conditions. Columns 1–4 include information for each experimental condition: NoExo, Free, Echo, and Pattern correspondingly while rows **A**–**C** show the knee kinematics (**A**), its phase portrait (**B**), and the foot contact with the floor (**C**). Information is represented for both legs: the paretic leg is represented in orange while the unassisted leg is represented in cyan. For the assistive experimental conditions, the exoskeleton set point is also represented in brown

**Fig. 13:**
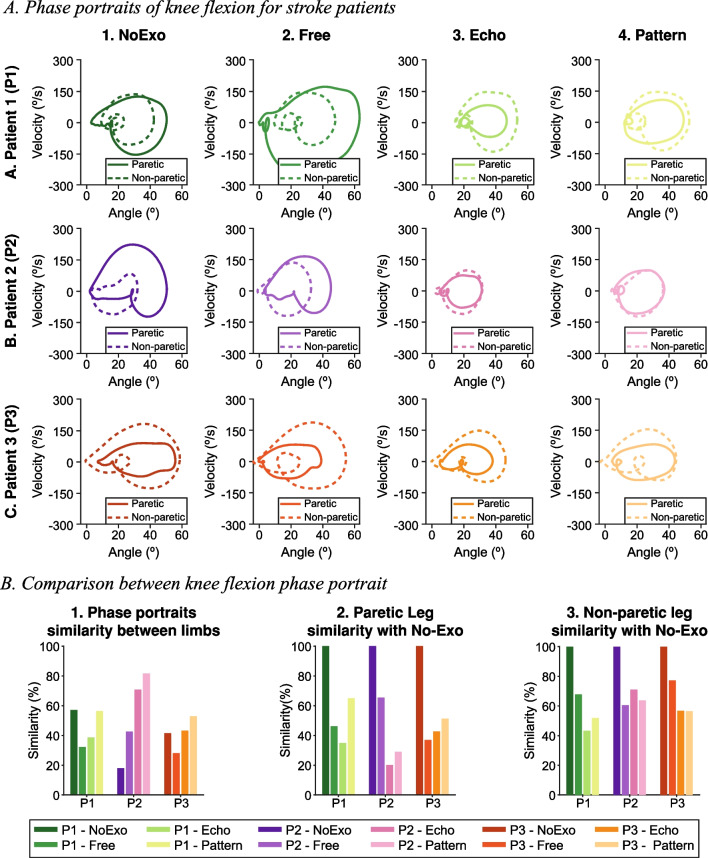
Knee flexion phase portraits for three stroke patients. **A** The median phase portrait for each limb, columns 1–4 include information for each experimental condition, while rows **A**–**C** show the data for the three patients. Solid lines are used for the impaired leg, while dashed lines are used for the nonparetic leg. **B** The similarity between phase portraits; Panel **B1** represents the similarity between both limbs under each experimental condition, and panels **B2**–**B3** represent the similarity between the movement of the current experimental condition and the movement during NoExo. Across the figure, data for the same patient (P) are represented by the same color (green for P1, purple for P2, and orange for P3) while the brightness indicates the experimental condition (from darkest to lightest: NoExo, Free, Echo, and Pattern)

During the third validation phase, we evaluated the performance of the prototype with real hemiparetic patients. To this end, we evaluated how the patients reacted to the assistance provided by the prototype; an example of the data recorded for a patient is represented in Fig. 12. To compare the motion dynamics in both knees, we used the median phase portrait of the flexion/extension movement (Fig. 13, Panel A). We evaluated their similarity according to Eq. (15), showing the results in Panel B of Fig. 13.

As shown in Panel B1 of Fig. 13, the robot assistance always improved the similarity between the motion of both limbs with respect to the motion in the Free condition. In two out of three patients (P1 and P3), wearing the robot (Free condition) hampered the symmetry in the motion; however, the action of the robot compensated for this effect (P1 during Pattern and P3 during Echo) or even increased the symmetry with respect to the NoExo condition (P3 during Pattern). In Subject P2, wearing the exoskeleton increased the symmetry, and the exoskeleton’s action even enhanced this improvement. In all subjects, the Pattern strategy led to a more symmetric motion than the Echo strategy.

Importantly, Panels B2 and B3 of Fig. 13 show the limb motion similarity with its movement during NoExo trials for the assisted and nonassisted legs, respectively. The motion of both legs changed due to the exoskeleton’s action and, therefore, the assistance not only acted to control the movement of the assisted leg but also affected the movement of the nonassisted leg to attain a more symmetric gait.

**Fig. 14:**
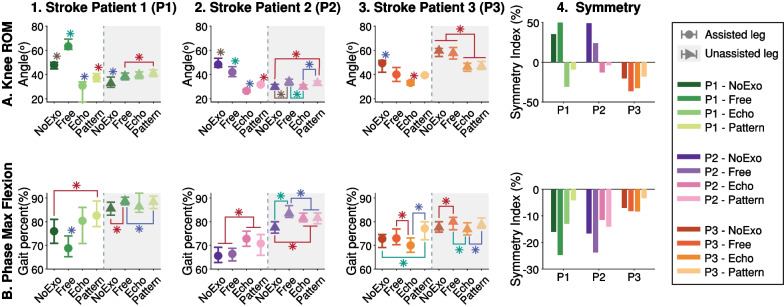
Knee kinematics symmetry for stroke patients. Rows **A** and **B** represent the knee range of motion and the phase at maximum flexion for the knee. Columns 1–3 represent the result for each subject; the markers indicate the median value, and the whiskers indicate the 10th–90th percentiles. Column 4 represents the symmetry index for each subject under each experimental condition; notice that symmetry indices closer to 0 indicate greater symmetry between limbs. Across the figure, identical colors represent the same patient (P) (P1 in green, P2 in purple and P3 in orange), the brightness represents the experimental condition (from lightest to darkest: No-Exo, Free, Echo and Pattern correspondingly) and the shape of the marker represents the assessed limb (circle for the assisted limb and triangle for the unassisted leg). Markers (*) show significant differences between experimental conditions within a limb of a subject

To evaluate the performance of the controllers, we evaluated the symmetry of the RoM and the phase of maximum knee flexion in both legs (Fig. 14) by using the symmetry index defined in the Eq. (16) (represented in the fourth column of Fig. 14). The robot’s action yielded more symmetric RoMs in all patients than the Free condition; in addition, pattern assistance and echo assistance in P1 and P2 attained a higher RoM symmetry even compared with the NoExo condition. The effect of the mass of the robot was heterogeneous: patients P1 and P3 worsened the RoM symmetry while P2 improved it. The symmetry of the maximum flexion phase decreased because of wearing the exoskeleton in all patients. However, the robot assistance compensated for this effect and improved the symmetry with respect to NoExo in all patients and under all control strategies, except P3 that remained unaffected during Echo.

**Fig. 15:**
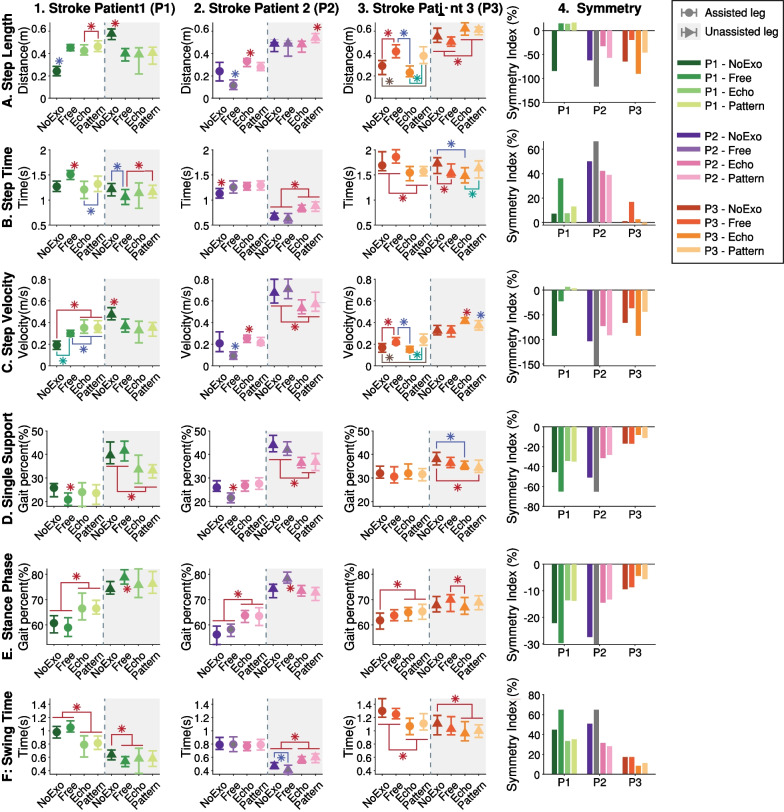
Gait features symmetry for stroke patients. Rows **A** to **F** include different gait metrics: step length (**A**), step time (**B**), step velocity (**C**), single-support duration (**D**), stance phase duration (**E**), and swing time (**F**). Columns 1–3 represent the result for each subject; markers indicate the median value and whiskers denote the 10th–90th percentiles. Column 4 represents the symmetry index for each subject under each experimental condition; notice that symmetry indices closer to 0 indicate a higher symmetry between limbs. Across the figure, identical colors represent the same patient (P) (P1 in green, P2 in purple and P3 in orange), the brightness represents the experimental condition (from lightest to darkest: No-Exo, Free, Echo and Pattern correspondingly) and the shape of the marker represents the assessed limb (circle for the assisted limb and triangle for the unassisted leg). Markers (*) show significant differences between experimental conditions within a limb of a subject

We also assessed the effect of the assistance on the compensation mechanisms developed by the patients. By using the floor contact information and the IMU data, we analyzed the influence of the exoskeleton on gait parameters related to compensatory strategies in hemiparetic gait such as (a) step length, (b) step time, (c) step velocity, (d) single-support duration, (e) stance phase duration, and (f) swing time (Rows A–F of Fig. 15).

We used the symmetry index to evaluate the action of the robot with respect to these metrics (fourth column of Fig. 15). Overall, the assistance provided by the robot led to more symmetric features compared with NoExo trials, or at least compensated for the detrimental effect of wearing the extra mass of the device. However, patients presented a heterogeneous response for some features.

Step length symmetry was improved during all assistive trials compared with the basal condition (excepting Echo in P3). However, each patient responded differently: improvements in P1 were because of wearing the robot (assistive strategies and Free condition yielded the same results), P3 improvements were also because of wearing the device, although the assistance decreased this symmetry (nonetheless, the Pattern condition supposed higher symmetry than the NoExo condition), and P2 reported symmetry improvements even when wearing the device resulted in its decrease (Panel 4A of Fig. 15).

For all patients, wearing the robot caused a decrease in the step time symmetry (Panel 4B of Fig. 15). However, this effect was compensated by the action of the assistive strategies, and P2 even increased step time symmetry with respect to his basal condition. Regarding the step velocity (Panel 4C of Fig. 15), both assistive strategies led to an increase of symmetry in P1 and P2 compared with the NoExo condition, although it was partially attained during the Free trial in P1; for P3, the Pattern assistance yielded the same symmetry improvement as the Free condition with respect to the NoExo trial, although the Echo assistance caused a decrease in the step velocity symmetry.

These improvements in step velocity symmetry occurred because all patients increased the step velocity of the assisted leg and reduced it in the nonassisted leg (P1 and P2) or increased it at a lower level (P3). The origin of these velocity changes was heterogeneous across patients, and the changes are related to the patient’s own strategies to cope with the robot’s actions. For instance, P1 increased the step length and reduced the step time in the paretic leg while reducing the step length of the nonparetic leg. P2 increased the step length and time in the paretic leg while maintaining step length and increasing the step time in the nonparetic leg. Finally, symmetry improvement in P3 was due to increasing step length and reducing step time in the paretic leg, and increasing the step length of the nonparetic leg.

Regarding symmetry of the stance and swing phases of the gait, the assistance provided by the exoskeleton always led to more symmetric single-support and stance step percentages compared to the NoExo condition (Panels 4D, E of Fig. 15), even when wearing the robot resulted in less symmetry than the basal condition (P1 and P2). Equally, the assistance provided by the robot improved the symmetry of swing time in all patients compared to NoExo, even when Free trials exhibited a decrease of symmetry in this metric (Panel 4F of Fig. 15).

All patients attained symmetry improvement in the single-support and stance phases by increasing their duration for the assisted leg and/or reducing these durations for the unassisted leg. Conversely, the strategy followed to improve symmetry in the swing time differed between subjects: P1 and P3 reduced swing time for both legs while P2 only increased it in the assisted leg.

## Discussion

This paper introduces the proof of concept of the REFLEX exoskeleton, which is aimed to serve as a tool to investigate the effects of unilateral assistance in hemiparetic gait. We present two controllers to provide this gait assistance based on the motion of the nonparetic leg of hemiparetic patients, searching a natural coordination between assisted and unassisted joints. Both controllers aimed to synchronize the generated assistance with the motion of the nonparetic leg motion of the patients: the Echo strategy replicated the movement of the unassisted leg over the assisted one, while the Pattern strategy synchronically applied a standard healthy pattern that was adapted to the range of motion of the healthy joint. We aimed to validate the assistance provided by these controllers from two perspectives: (1) the generation of the assistive pattern and (2) the effects of this assistance on the users’ gait, focusing mainly on the compensation mechanisms developed by hemiparetic patients to attain a functional gait.

### On assistance generation

The basis of both assistive strategies is an AO that estimates the gait phase of the unassisted leg in real-time. Since this estimation is used to synchronize the assistance application, we evaluated AO’s performance and its reaction to speed changes. Trials with healthy subjects at variable gait speed resulted in gait phase estimations with RMS errors lower than 2.4. According to Ruiz-Garate et al., we can consider that the phase estimation is synchronized with gait if the error is lower than 10% [65]; thus, our approach is valid to coordinate the assistance provided by the prototype. Our results are in the range of those previously published by other authors, who reported RMS phase estimation errors of 3% using noncontact capacitive sensors [66], 2% using insole pressure sensors to measure the vertical ground reaction force [67], or 1.4% using an encoder to measure the hip angle [67].

During VariableSpeed trials, we also evaluated the gait frequency estimation while changing walking velocity. The low RMS error in frequency estimation (lower than 0.02 Hz) highlights the correct adaptation of the AO and confirms that it is able to handle naturally varying gait speed.

Since the controllers aim to generate an assistive pattern based on the motion of the unassisted leg, we simulated the performance of the controllers and compared the dynamics of the assistance with the gait patterns of healthy subjects. The results indicated that both strategies attained similarities with the master leg exceeding 75%. However, the average similarity for the Echo strategy (84.3%) was significantly higher than that achieved by the Pattern strategy (77.5%). Both strategies were also affected differently by changes in velocity. For the Echo strategy, the similarity was higher during the constant speed trials (87%) than during the variable speed trials (82%). In comparison, the Pattern strategy did not suppose significant differences between these trials (77% and 78.6% during variable and constant speed trials, respectively).

Concerning the timing of the assistance, the reference patterns and the motion of the equivalent legs of healthy subjects showed high synchrony. Both controllers attained a high average correlation for these movements exceeding R = 0.9 in both cases, although the value was significantly higher for the Echo strategy (R = 0.95) than for the Pattern strategy (R = 0.9). Speed changes also affected these strategies differently. Echo correlation significantly decreased during variable gait speed trials (R = 0.94) compared to constant gait speed trials (R = 0.98), while we did not identify significant changes in Pattern strategy due to speed variability.

Delays between the assistive pattern and the motion of the equivalent leg also pointed to highly coordinated movements. The average RMS delay of the instant of maximum knee flexion remained lower than 70 ms, which is lower than the reaction time of voluntary muscle contractions (180 ms) and, therefore, it is valid for the control of robotic exoskeletons [68, 69].

**Fig. 16:**
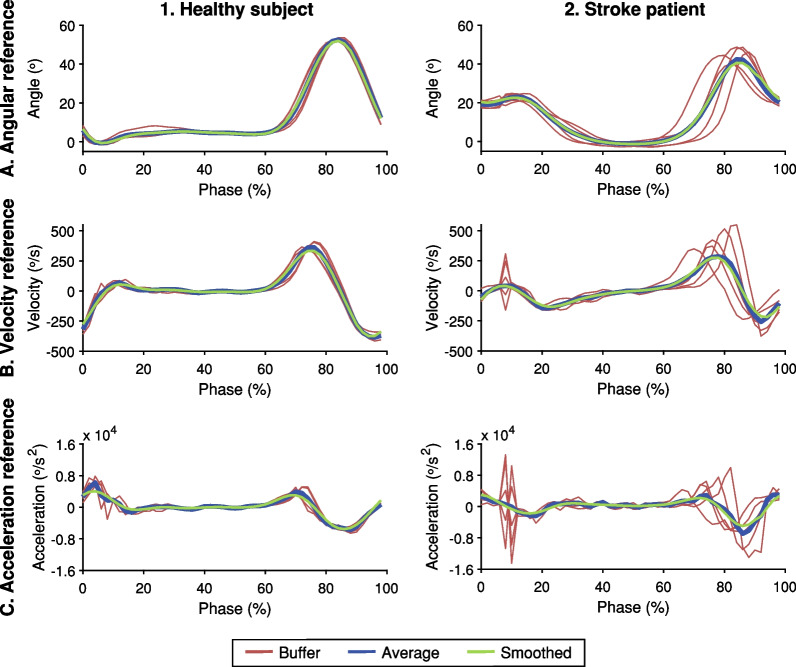
Examples of the three references generated by the Echo strategy (angle, velocity, and acceleration for rows **A**–**C**) during trials with a healthy subject (column 1) or a stroke patient (column 2). Red lines represent the content of the five steps buffer, the blue line is the average step, and the green line is the smoothed average step that serves as the pattern reference

The better performance of the Echo strategy, in terms of similarity with the master motion and correlation with the equivalent motion, was due to the controller’s working principle, which directly replicated the average motion of the master leg. However, because of the five-step buffer, this controller assumed that the pattern of the master leg is repetitive between cycles. Changes in gait speed affect this repetitiveness [70], explaining the different performance between variable and constant gait speed trials. In stroke patients, this repetitiveness decreased with respect to healthy subjects [71]; thus, in these patients, the performance of this controller worsened compared with the performance in healthy subjects (see Fig. 16).

### On gait adaptation to assistance

The results indicated that healthy subjects decreased the similarity between the motion of both legs because of wearing the exoskeleton’s extra load. Although robot assistance partially counteracted this effect, the device could not attain the symmetry these subjects showed during normal walking. Interestingly, the symmetry improvements yielded by the exoskeleton action were not only due to changes in the assisted leg; rather, the motion of the nonassisted leg also evolved to attain a higher level of symmetry. Therefore, we can conclude that healthy subjects reacted to the assistance provided by the robot by actively changing their own gait pattern to attain a more symmetric gait. This may be justified by the influences of the proprioceptive changes produced by the exoskeleton action on the gait pattern generators [72, 73].

The effect of the assistance on hemiparetic gait was homogeneous. All subjects increased the similarity between the motion of both limbs due to the provided assistance compared with the Free condition. However, despite these improvements, the response with respect to the basal condition differed between subjects. Three out of six experimental conditions improved the symmetry with respect to the basal condition, two out of six compensated for the hampering effect of wearing the robot, and only one out of six could not fully compensate for it.

Notably, the Pattern strategy achieved better results than the Echo strategy in stroke patients. Under the Pattern strategy, two out of three patients improved the motion symmetry with respect to their basal conditions, and only one subject compensated for the effect of wearing the robot. Conversely, under the Echo strategy, only one subject improved with respect to his basal condition, one subject compensated for the effect of wearing the device, and one subject could not compensate for this effect. As previously mentioned, this worse performance was due to the low repetitiveness of the nonparetic limb motion that affected the Echo more than the Pattern strategy.

Compared with the Free condition, the assistance results were similar for both kinds of subjects (healthy and hemiparetic), as the assistance increased the similarity of the motion, the RoM, and the phase of maximum flexion of the knee joint. However, they differed in comparing the results with their basal condition. While the robot was not able to improve the gait symmetry in healthy subjects, it was able to assist the gait of hemiparetic subjects to improve it.

The assistance provided by the robot also led to more symmetric gait features in stroke patients. The Pattern strategy improved the step length, time and velocity symmetry in all patients with respect to their basal condition, or, at least, it compensated for the hampering effect of wearing the robot. However, the Echo strategy could not achieve these improvements in one out of the three hemiparetic patients. Similarly, robotic assistance led to more symmetric single-support, stance phases, and swing times. In these cases, both assistive strategies achieved similar results.

These results indicate that the device’s action can assist the gait in hemiparetic patients in such a way that classic compensations to attain a functional gait are decreased. In this sense, the robot’s action increased the stance phase in the paretic leg and reduced the single-support phase in the nonparetic leg, compensating for typical disturbances in hemiparetic gait [74]. The assistance provided by the exoskeleton also yielded a more symmetric step length, time, and velocity. These improvements could be associated with decreased asymmetric limb loading and gait inefficiency [15] and can improve patients’ balance during walking [75].

Notably, these symmetry improvements are not only due to changes in the assisted limb. In contrast, the motion of the unassisted leg of the patients also evolves to attain a more symmetric gait globally. As REFLEX’s action assists the impaired limb, fewer compensatory actions are required from the nonparetic leg, and therefore, its motion is also improved. Patients seemed to integrate feedback from the assisted leg to adjust the action of the nonassisted limb, improving gait performance and stability [76]. This may be explained by the action of central pattern generators located in the spinal cord using feedback from proprioceptive muscular and tendinous receptors informing the brain of applied loads and forces due to the exoskeleton action [73]. Although cerebellar participation in gait pattern generation has not been clearly elucidated, it may be partly responsible for the compensation seen in our study as none of the subjects had cerebellar impairments [77, 78].

This assistance integration could release the nonparetic leg from excessive loading patterns that may lead to secondary musculoskeletal complications [79], since temporal asymmetry correlates with increased vertical ground reaction forces in the nonparetic leg [80]. Interestingly, these adaptations in the nonassisted leg were attained in a short time (5 min), and they occurred naturally without following any kind of instruction. Adaptations in the nonassisted leg were also reported by Chinimilli et al. [81], who found that unilateral knee assistance led to an increase in the stability of the unassisted leg due to interlimb coordination in healthy subjects. For stroke patients, adaptations in the nonassisted leg were also reported during early robotic rehabilitation with the single-leg version of the HAL exoskeleton [82].

Our results on gait symmetry improvements are similar to those previously reported by other authors who aimed to unilaterally assist impaired gait. For example, Arazpour et al. described an active KAFO to improve symmetry in poliomyelitis subjects based on an FSM controller and reported symmetry improvements in swing time, stance phase percentage, and knee flexion during swing [64]. Similarly, Beyl et al. reported timing symmetry improvements in one multiple sclerosis patient when assisted by the KNEXO prototype [83]. The unilateral exoskeleton robot developed by Shenzhen University [84] enabled improvements in the range of motion of the joints of three hemiparetic subjects, although their controller was based on detecting the heel-strike event to trigger the assistive action.

Other previous approaches, such as REFLEX, were also based on the motion of the nonparetic leg to coordinate the action of robotic exoskeletons. For example, Complementary Limb Motion Estimation presented promising results with healthy subjects [37, 38], but it was not tested with stroke patients. Kawamoto et al. described a control strategy based on the kinematics of the nonparetic leg, similar to our Echo control, that reported significant symmetry improvements in one stroke patient while the HAL exoskeleton assisted his gait [21]. However, in contrast with our approach, the control paradigm of these examples was based on the detection of gait events, and therefore, they were not adaptive in real-time to changes in gait frequency.

In contrast, the ALEX III prototype used a pool of AOs and nonlinear filters to reconstruct the joint patterns of the nonparetic leg that acted as references for the hip and knee of the paretic limb [85]. They tested this approach in three healthy subjects simulating impairments in one leg by wearing a load on the ankle and reported gait symmetry improvements due to the action of the device. Although they reported symmetry improvements, with our approach, we assessed that the origin of these improvements is twofold, resulting from both the assistance of the paretic leg and the adaptation of the nonassisted leg.

In contrast to previous studies, we compared the performance of assisting the paretic leg with the kinematics of the nonparetic leg or with a standard healthy pattern. Considering the global results with stroke patients, the Pattern strategy seemed to perform better than the Echo strategy. This suggests that providing assistance according to a normalized healthy pattern would lead to better outcomes than assisting with the motion of the nonparetic leg of the subject. As we have attained changes in the motion of both limbs, assisting the paretic limb with a healthy motion could lead to the conclusion that the nonparetic leg would also improve its motion to a healthier pattern without compensation mechanisms.

Hypothetically, this approach might lead to improvements in gait beyond compensatory strategies arising from hemiparesis, promoting new functional patterns with lower energetic cost. This would imply that users integrated the robot’s action, thus affecting their own gait patterns and pointing out that the robotic exoskeleton was embodied at a certain level. This should be explored in subsequent studies with larger sample sizes to evaluate its feasibility as an assistive strategy and its rehabilitation potential. In the same way, the REFLEX prototype could be extended by adding active joints at the hip or ankle to increase the assistance provided to the user and evaluate the effect of other assistive strategies, such as assisting ankle push-off or hip contribution to forward propulsion.

## Conclusions

This paper presented the REFLEX prototype and its control algorithms to evaluate the effects of unilateral assistance in hemiparetic gait. REFLEX is an active KAFO that assists the knee flexion/extension of the impaired leg of stroke survivors. The control paradigm that we followed is based on the fact that the motion of both legs is shifted 180º, so we calculated the desired gait phase of the paretic leg by estimating the real-time gait phase of the nonparetic leg.

We also proposed two control strategies to assist the movement of the paretic limb. The Echo control strategy aimed to directly replicate the movement of the nonparetic leg, while the adaptive healthy pattern strategy aimed to synchronically apply a standard healthy kinematic pattern to assist the movement. These two strategies promote a natural synchronization between assisted and unassisted joints.

Experimental results with three healthy subjects and three stroke survivors revealed that the REFLEX prototype is able to assist the gait of hemiparetic subjects in coordination with the movement of the nonparetic leg. The patients showed improved spatial and temporal symmetry due to the assistance provided by the exoskeleton. These improvements were related not only to the motion guidance imposed by the robotic exoskeleton but also to the adaptation that occurred in the nonassisted limbs, which seemed to decrease their compensatory strategies.

## Data Availability

The datasets generated and/or analyzed during the current study are available from the corresponding author on reasonable request.

## Abbreviations

AO: Adaptive frequency oscillator
Echo: Trial during which the exoskeleton assisted the user according to the Echo-control assistive strategy
EMG: Electromyography
Free: Trial during which the subjects wore the exoskeleton, but it was mechanically decoupled from the actuator, enabling the joint’s free movement
FSM: Finite State Machine
HS: Healthy subject
KAFO: Knee-ankle–foot orthosis
NoExo: Trial during which the subject was not wearing the exoskeleton
Pattern: Trial during which the exoskeleton assisted the user according to the adaptive healthy pattern assistive strategy
RoM: Range of motion
VariableSpeed: Trial during which the healthy subject walked at variable gait speed
